# Integrating social and cardiometabolic risk factors in predicting cardiometabolic multimorbidity: a Bayesian model development and internal validation in Swedish middle-aged adults

**DOI:** 10.1038/s41598-026-67901-3

**Published:** 2026-08-25

**Authors:** Kanya Anindya, Juan Merlo, Lars Lind, Tomas Jernberg, Lars Weinehall, Maria Rosvall, Marcus Bendtsen, Nawi Ng

**Affiliations:** 1 School of Public Health and Community Medicine, Institute of Medicine, Sahlgrenska Academy, University of Gothenburg, Guldhedsgatan 5A, Plan 3, 413 20 Gothenburg, Sweden; 2 Unit for Social Epidemiology, Department of Clinical Sciences, Faculty of Medicine, Lund University, Malmö, Sweden; 3 Centre for Primary Health Care Research, Region Skåne, Malmö, Sweden; 4 Department of Medical Sciences, Uppsala University, Uppsala, Sweden; 5 Department of Clinical Sciences, Danderyd Hospital, Karolinska Institute, Stockholm, Sweden; 6 Department of Epidemiology and Global Health, Umeå University, Umeå, Sweden; 7 Social Medicine, Regionhälsan, FoUUI, Region Västra Götaland, Sweden; 8 Department of Health, Medicine and Caring Sciences, Linköping University, Linköping, Sweden

**Keywords:** Risk score, Bayesian, Cardiometabolic disease, Cardiovascular disease, Multimorbidity, Biomarkers, Cardiology, Diseases, Health care, Medical research, Risk factors

## Abstract

Cardiometabolic diseases often cluster as multimorbidity, yet current risk prediction models typically focus on a single disease and rarely incorporate social determinants. This study aims to develop a Bayesian risk prediction model for cardiometabolic multimorbidity in middle-aged Swedish adults. This prospective cohort included 11,964 adults without prior cardiometabolic disease from the Swedish CArdioPulmonary bioImage Study (SCAPIS; enrolment 2013–2016). Cardiometabolic disease was defined as incident type 2 diabetes (T2D), ischaemic heart disease/heart failure, or stroke. Regularised Bayesian logistic regression models were developed to predict any cardiometabolic disease (≥ 1 condition) and cardiometabolic multimorbidity (≥ 2 conditions) within 2–5 years. Twenty-two social and cardiometabolic risk predictors were considered. During follow-up, 6.0% of participants developed cardiometabolic disease(s) (5.7% with a single cardiometabolic disease and 0.3% with multimorbidity). The prediction model showed fair discrimination for any cardiometabolic disease (AUC 0.76, 95% CI 0.74–0.78) and good discrimination for multimorbidity (AUC 0.89, 95% CI 0.86–0.93). The highest predicted risks were observed among older foreign-born males with hypertension, low HDL-C, high waist circumference, and current/ex-smoker, with predicted risks of 34.7% (95% CrI 30.3%–39.5%) for any cardiometabolic disease and 4.4% (95% CrI 2.0%–7.9%) for multimorbidity. These findings suggest that social determinants and uncertainty estimates may provide useful information for cardiometabolic disease prediction, though further validation is needed before use in clinical practice.

**Supplementary Information:**

The online version contains supplementary material available at 10.1038/s41598-026-67901-3.

## Introduction

Cardiometabolic disease comprises interconnected conditions, including type 2 diabetes (T2D) and cardiovascular complications, such as ischaemic heart disease and stroke, which are the leading causes of death and disability globally^1–3^. These conditions share pathophysiological mechanisms, including insulin resistance, chronic inflammation, and endothelial dysfunction^2,4^, and often cluster as cardiometabolic multimorbidity, defined as the presence of at least two cardiometabolic diseases in an individual^5^. In Sweden, evidence from the Swedish CArdioPulmonary bioImage Study (SCAPIS) indicates that 24.4% of adults aged 50–64 years had one cardiometabolic disease and 2.8% had cardiometabolic multimorbidity, with social patterning by sex and education^6^.

One of the main challenges in managing cardiometabolic disease is the prolonged subclinical phase, during which metabolic and vascular changes accumulate before clinical diagnosis^2^. This latent progression underscores the need for prediction tools (‘risk scores’) to identify individuals at elevated risk and support timely primary prevention^7^. Existing cardiovascular risk scores, including the Framingham Risk Score^8^ and Systematic COronary Risk Evaluation (SCORE) models^9^, are valuable but typically focus on single disease outcomes, overlooking the growing burden of cardiometabolic multimorbidity. While the American Heart Association emphasises the importance of social determinants for risk prediction^10^, existing scores continue to rely largely on clinical risk factors^8,9,11–15^ and rarely incorporate social determinants^10,16–18^. Previous studies have applied machine learning approaches to improve discrimination; however, these methods can be challenging to interpret and often do not provide uncertainty measures around individual risk estimates^7,19,20^.

Incorporating both social and cardiometabolic risk factors may improve the comprehensiveness and interpretability of risk prediction models and help identify high-risk groups needing intensified prevention or treatment in both population and clinical settings. Bayesian approaches can also quantify uncertainty around individual risk estimates using credible intervals, supporting more interpretable risk communication^21^. This study aims to develop and internally validate Bayesian prognostic models for incident cardiometabolic disease and cardiometabolic multimorbidity, incorporating social and cardiometabolic risk factors, in middle-aged Swedish adults with no prior cardiometabolic disease.

## Materials and methods

### Study population

This prospective cohort study used data from SCAPIS, a population-based cohort that randomly invited 59,909 individuals aged 50–64 years living in six Swedish university municipalities (Gothenburg, Linköping, Malmö/Lund, Stockholm, Umeå and Uppsala) between November 2013 and December 2018^22^. 30,154 out of 59,909 individuals (50.3%) participated. SCAPIS were linked to the National Patient Register (NPR) for outpatient (1997–2018) and inpatient care (1970–2018), as well as the Swedish Prescribed Drug Register (SPDR, 2005–2020). Individuals who consented to link their responses to the register data were considered in the analysis (*n* = 30,030).

We restricted the cohort to participants recruited in SCAPIS between 1 November 2013 and 31 December 2016 (*n* = 16,266). This timeframe was chosen to allow for a longer follow-up period for outcome assessment, as register data were available only up to 31 December 2018. This study further excluded participants with a history of T2D, heart disease (defined as ischaemic heart disease, heart failure), coronary revascularisation, or stroke at baseline (*n* = 1,783). These conditions were identified using self-reported information, NPR, SPDR (for T2D), and glycaemic status at baseline SCAPIS examination (for T2D). The total eligible sample comprised 14,483 participants, of whom 11,964 (82.6%) had complete responses for all variables. A flowchart of participant selection and missing data is available in Supplementary Fig. A1 and Supplementary Table A1. The study is reported according to the Transparent Reporting of a Multivariable Prediction Model for Individual Prognosis or Diagnosis + Artificial Intelligence guideline^23^.

### Variables

#### Outcomes

Cardiometabolic disease was defined as incident T2D, heart disease (ischaemic heart disease and heart failure), or stroke after baseline. Outcomes were identified using the NPR for diagnoses and coronary revascularisation, and the SDPR for glucose-lowering medications. The International Classification of Diseases (ICD), 10th edition, codes were: E11–E14 for T2D, I20–I25; and I50 for ischaemic heart disease and heart failure; and I60, I61, I63 and I64 for stroke^1^. Coronary revascularisation was identified using procedure codes FNA, FNB, FNC, FND, FNE, and FNG. Glucose-lowering medications were identified using ATC codes A10A and A10B, excluding individuals with type 1 diabetes. We derived two binary outcomes: any cardiometabolic disease (at least one cardiometabolic disease) and cardiometabolic multimorbidity (two or more cardiometabolic diseases). This definition captures only the occurrence of multiple cardiometabolic diagnoses, rather than disease progression or temporal disease trajectories.

#### Predictors

Predictors were selected based on prior studies and the availability of variables in SCAPIS^9,12,13,24^. Social risk factors (defined in this study as sociodemographic characteristics and family background) included sex (female, male), age (in years), education (tertiary/higher, secondary/lower), difficulty in managing expenses (no, yes), participants’ country of birth (Swedish-born, foreign-born), type of residency (urban, rural), site of examination (Göteborg, Linköping, Malmö, Stockholm, Umeå, Uppsala) and parental history of cardiometabolic disease (no, yes).

Behavioural, psychosocial, and metabolic risk factors were assessed at baseline. Behavioural predictors included self-reported smoking status (non-smoker, current/ex-smoker), hazardous alcohol consumption (the Alcohol Use Disorders Identification Test/AUDIT score ≥ 8)^25^, sodium and fibre intake (g/day) from the food frequency questionnaire, physical inactivity based on accelerometery (< 75 min vigorous activity/week and sedentary time at least 9.5 h/day)^26^, and sleep duration (7–8 h, < 7 or ≥ 9 h)^27^. Psychosocial predictor was assessed as psychosocial stress, defined as persistent feelings of tension, irritability, anxiety, or difficulty sleeping attributable to work or home conditions, experienced during the past year or longer^28^.

Metabolic risk factors were assessed using blood sample analyses and records of prescribed medications at or before the baseline interview. Systolic blood pressure (SBP, in mmHg) and diastolic blood pressure (DBP, in mmHg), waist circumference (in cm), high-density-lipoprotein-cholesterol (HDL-C, in mg/dL) and non-HDL-cholesterol (non-HDL-C, in mg/dL) were included as continuous variables^12^. Blood pressure- and lipid-lowering medication were included as binary predictors (no, yes).

To adjust for unequal follow-up, we included follow-up duration (in days), defined as the number of days from the interview date to 31 December 2018, the last date of available register data. Details on the predictor measurements are available in Supplementary Table A2.

### Statistical analyses

Baseline characteristics were summarised using descriptive statistics. Incident cardiometabolic disease and cardiometabolic multimorbidity were predicted using Bayesian logistic regression. A Bayesian framework was used because it yields full posterior distributions for parameters and predicted risks, supporting direct uncertainty quantification and offering advantages for complex models and low event rates^29,30^.

For each outcome, we fitted two models:

- *Model 1 (unregularised)* Bayesian logistic regression was estimated with weakly informative Cauchy priors on the coefficients (centre 0, scale 2.5), following Gelman et al.^30^. To account for the low outcome incidence, the intercept prior was specified separately for each outcome, using a weakly informative Gaussian prior with mean equal to the log-odds of the observed outcome proportion and standard deviation 2.5.
- * Model 2 (regularised)* A Bayesian logistic regression model was estimated with independent double exponential (Laplace) priors assigned to the regression coefficients to induce shrinkage. The global shrinkage parameter, τ, followed a weakly informative half-Cauchy hyperprior (centre 0, scale 0.50). The intercept used the same prior as in Model 1 (unregularised). This approach performs a type of variable selection and may enhance predictive performance through regularisation.

Models were fitted using Markov Chain Monte Carlo (MCMC) sampling with four chains and 2000 iterations, and convergence was assessed using the Gelman-Rubin diagnostic ($\:\widehat{R}$ < 1.05) and effective sample sizes (ESS > 400).

Predictive performance was internally validated using 10-fold cross-validation, which is recommended by a previous study when data is limited, e.g., small event^31^. In each iteration, one-fold was used as the test set and the remaining nine as the training set. Within each fold, continuous predictors were standardised using the mean and standard deviation (SD) in the training fold, and the same values were applied to the test fold. During cross-validation, the intercept prior was centred using the outcome proportion in the training fold to avoid using information from the test fold. Calibration was evaluated using calibration plots and the calibration intercept and slope. Discrimination was assessed using the area under the receiver operating characteristic curve (AUC) and the true positive rate (TPR) at a prespecified false positive rate (FPR) of 10% (TPR_FPR10%_). AUC summarises discrimination between individuals with and without the event. TPR_FPR10%_ represents the proportion of true cases detected when the false-positive rate is fixed at 10%, corresponding to a specificity of 90%^32^.

To assess the contribution of different predictor blocks, we fitted nine prespecified blockwise regression models and compared the AUCs^28^. Blocks included: (A) age; (B) sex; (C) age & sex; (D) social; (E) behavioural & psychosocial; (F) metabolic factors; (G) social, behavioural & psychosocial; (H) age, sex, parental history of CMD, behavioural, psychosocial, and metabolic factors (except socioeconomic and geographical factors); and (I) full model including all predictors. Incremental discrimination was calculated as the change in cross-validated AUC (ΔAUC) relative to the age-only model as the reference. To complement AUC-based discrimination, we further assessed the incremental clinical utility of socioeconomic and geographical factors by comparing Model H against the full model (Model I) using decision curve analysis, net reclassification improvement (NRI), and integrated discrimination improvement (IDI). We examined potential non-additive effects by adding five a priori interaction terms to the full model, including: (A) age × sex; (B) country of birth × education; (C) sex × HDL-C; (D) smoking × non-HDL-C; (E) waist circumference × non-HDL-C. These interactions were selected based on clinical and epidemiological plausibility^13,33–35^ and were limited in number to reduce the risk of overfitting.

The best performing model (Model 1 or Model 2) was refitted in the full dataset to obtain posterior median odds ratios (ORs) and 95% credible intervals (CrI). Individual predicted probabilities and their 95% CrI were derived from the posterior predictive distribution. Risk stratification matrices were further constructed using clinically meaningful cut-offs for selected key predictors, informed by the ORs.

Four sensitivity analyses were conducted. *First*, we performed multiple imputation by chained equations using fully conditional specification (*m* = 20) (*mice* package). *Second*, we evaluated performance using Bayesian Additive Regression Trees (BART) to capture non-linearities and higher-level interactions without pre-specification with 10-fold cross-validation (*dbarts* package)^36^. *Third*, because the primary outcome combined several cardiometabolic diseases (T2D, ischaemic heart disease, stroke, and heart failure), we also fitted two disease-specific models: (1) T2D and (2) heart disease and stroke. The same predictors, priors, and 10-fold cross-validation were used as in the main analysis, and ORs (95% CrI) and discrimination (AUC and TPR_FPR10%_) were compared with the primary model. *Fourth*, to contextualise predictive performance against an established cardiovascular risk score, SCORE2 was applied to participants with complete SCORE2 predictors^9^. The SCORE2 algorithm, based on age, sex, current smoking, SBP, total cholesterol, and HDL-C, was used with moderate-risk regional recalibration corresponding to Sweden’s risk region^9,37^. Fixed coefficients were applied without refitting, and discrimination was compared with the full model for heart disease or stroke, which approximates the main non-fatal cardiovascular endpoints captured by SCORE2. Exploratory comparisons were also performed for any cardiometabolic disease and multimorbidity.

All statistical analyses were performed using R (version 4.4.1) with Stan (version 2.32.7)^38^.

## Results

Among the 11,964 participants, the mean age was 56.9 years (SD 4.3), and 54.1% were females (Table 1). Most participants were born in Sweden, and among those born outside Sweden, 40.0% were from non-European countries. During a median follow-up of 2.92 years, 5.7% developed a single cardiometabolic disease, and 0.3% had cardiometabolic multimorbidity. Among participants who developed cardiometabolic multimorbidity, the most common sequence was heart disease preceding T2D (H → D; *n* = 21), followed by T2D preceding heart disease (D → H; *n* = 11) (Supplementary Table A4). The recorded interval between the first and second diagnoses was more than one year in 50.0% of multimorbidity cases, while 15.0% had diagnoses dates within one month (Supplementary Table A5).

**Table 1:** Participants’ characteristics.

Variables	*n* (%)
N	11,964 (100.0)
Age, mean (SD)	56.9 (4.3)
Sex	
Female	6478 (54.1)
Male	5486 (45.9)
Family history of cardiometabolic disease	
No	5601 (46.8)
Yes	6363 (53.2)
Educational attainment	
Tertiary	5538 (46.3)
Secondary/lower	6426 (53.7)
Financial difficulty	
No financial difficulty	11,339 (94.8)
Financial difficulty	625 (5.2)
Country of birth	
Swedish-born	10,044 (84.0)
Foreign-born^1^	1920 (16.0)
Residency	
Urban	11,263 (94.1)
Rural	701 (5.9)
Site of examination	
Gothenburg	3685 (30.8)
Linköping	1646 (13.8)
Malmö	3097 (25.9)
Stockholm	2031 (17.0)
Umeå	233 (1.9)
Uppsala	1272 (10.6)
Smoking	
Non-smoker	5885 (49.2)
Current/ex-smoker	6079 (50.8)
Alcohol consumption	
Abstinence/low-risk consumption	10,751 (89.9)
Hazardous consumption/alcohol dependence	1213 (10.1)
Sodium intake (gram/day), median (IQR)	2.1 (1.6–2.7)
Fibre intake (gram/day), median (IQR)	17.9 (12.5–24.9)
Physical activity	
Low risk	8995 (75.2)
At risk: highly sedentary & low VPA	2969 (24.8)
Self-perceived stress	
No/several periods	9503 (79.4)
Constant	2461 (20.6)
Sleep duration	
7–8 h	7319 (61.2)
<7 or ≥ 9 h	4645 (38.8)
SBP (mmHg), mean (SD)	125.0 (17.3)
DBP (mmHg), mean (SD)	76.4 (10.8)
Waist circumference (cm), mean (SD)	93.3 (12.4)
HDL-C (mg/dL), median (IQR)	61.8 (50.2–77.3)
Non-HDL-C (mg/dL), median (IQR)	147.0 (123.7–174.0)
Blood pressure lowering medication	
No	7683 (64.2)
Yes	4281 (35.8)
Lipid-lowering medication	
No	10,721 (89.6)
Yes	1243 (10.4)
Follow-up duration (in days), median (IQR)	1069 (874–1349)
Number of cardiometabolic disease	
No cardiometabolic disease	11,242 (94.0)
Single cardiometabolic disease	682 (5.7)
Cardiometabolic multimorbidity	40 (0.3)

DBP: diastolic blood pressure; HDL-C: high-density lipoprotein cholesterol; IQR: interquartile range; SBP: systolic blood pressure; SD: standard deviation; VPA: vigorous physical activity. Continuous variables are presented as mean (SD) or median (IQR) for skewed distributions. Categorical variables are presented as n (%).

^1^Of the 1,920 foreign-born participants, 20.0% were from Nordic countries (except Sweden), 40.0% from other European countries, and 40.0% from non-European countries. Nordic countries include Norway, Denmark, Finland, and Iceland. Country of birth were treated as binary in the analysis due to small number of samples in each category.

### Predictive performance and model selection

Predictive performance was assessed using calibration plots and discrimination metrics, including cross-validated AUC and TPR_FPR10%_, comparing the *unregularised model* (Model 1) and *regularised model* (Model 2).

For any cardiometabolic disease, both models were well calibrated, with slightly better calibration for Model 2 (intercept − 0.01 [95% CI -0.22–0.21], slope 0.99 [95% CI 0.91–1.07]) than Model 1 (intercept − 0.12 [95% CI -0.32–0.09], slope 0.94 [95% CI 0.86–1.02]) (Supplementary Fig. A2, A–B). For cardiometabolic multimorbidity, Model 1 was poorly calibrated (intercept − 1.10 [95% CI -1.70–-0.50], slope 0.70 [95% CI 0.53–0.86]), while Model 2 showed improved calibration (intercept 0.27 [95% CI -0.33–0.87], slope 1.02 [95% CI 0.79–1.25]), although the confidence intervals were wide due to the small number of events (Supplementary Fig. A2, C–D).

Discrimination (Table 2) was identical for any cardiometabolic disease (AUC 0.76), with Model 2 showing a small gain in TPR_FPR10%_. For cardiometabolic multimorbidity, Model 2 had higher AUC (0.89 vs. 0.87) and TPR_FPR10%_ (0.75 vs. 0.58), although these estimates were based on a small number of observed multimorbidity events. The TPR_FPR10%_ of 0.75 in Model 2 means that the model correctly identifies 75% of true cases while misclassifying only 10% of non-cases as positive.

**Table 2:** Predictive performance of unregularised and regularised Bayesian models and blockwise regression for any cardiometabolic disease and multimorbidity.

Models	AUC (95% CI)	△ AUC^6^	TPR_FPR 10%_
Any cardiometabolic disease (*N* = 11,964)			
Model 1: Unregularised Bayesian logistic regression^1^	0.76 (0.74–0.78)		0.37
Model 2: Regularised Bayesian logistic regression^2^	0.76 (0.74–0.78)		0.38
Blockwise regressions based on Model 2^3^			
(A) Age	0.62 (0.60–0.65)	Ref.	0.19
(B) Sex	0.64 (0.62–0.66)	+ 0.02	0.22
(C) Age & sex	0.67 (0.65–0.69)	+ 0.05	0.26
(D) Social factors	0.70 (0.68–0.72)	+ 0.08	0.28
(E) Behavioural & psychosocial factors	0.61 (0.59–0.63)	-0.01	0.18
(F) Metabolic factors	0.74 (0.72–0.75)	+ 0.12	0.34
(G) Social, behavioral & psychosocial factors	0.70 (0.68–0.72)	+ 0.08	0.30
(H) Age, sex, parental history, behavioural, psychosocial, and metabolic factors⁴	0.74 (0.73–0.77)	+ 0.12	0.35
(I) Full model^5^	0.76 (0.74–0.78)	+ 0.14	0.38
Cardiometabolic multimorbidity (*N* = 11,964)			
Model 1: Unregularised Bayesian logistic regression^1^	0.87 (0.83–0.92)		0.58
Model 2: Regularised Bayesian logistic regression^2^	0.89 (0.86–0.93)		0.75
Blockwise regressions based on Model 2^3^			
(A) Age	0.73 (0.64–0.81)	Ref.	0.38
(B) Sex	0.75 (0.66–0.84)	+ 0.02	0.43
(C) Age & sex	0.78 (0.71–0.86)	+ 0.05	0.50
(D) Social factors	0.79 (0.72–0.86)	+ 0.06	0.45
(E) Behavioural & psychosocial factors	0.74 (0.67–0.81)	+ 0.01	0.28
(F) Metabolic factors	0.88 (0.84–0.92)	+ 0.15	0.65
(G) Social, behavioral & psychosocial factors	0.81 (0.74–0.87)	+ 0.08	0.60
(H) Age, sex, parental history, behavioural, psychosocial, and metabolic factors⁴	0.89 (0.86–0.92)	+ 0.16	0.75
(I) Full model^5^	0.89 (0.86–0.93)	+ 0.16	0.75

AUC: area under the receiver operating curve (estimated based on 10-fold cross-validation); CI: confidence interval; TPR: true positive rate; FPR: false positive rate; TPR_FPR10%_: true positive rate at a 10% false positive rate.

All predictors: age (in years), sex, family history of cardiometabolic disease, educational attainment, financial difficulty, country of birth, residency, site of examination, smoking, alcohol consumption, sodium intake (gram/day), fibre intake (gram/day), physical activity, self-perceived stress, sleep duration, SBP (mmHg), DBP (mmHg), waist circumference (cm), HDL-C (mg/dL), non-HDL-C (mg/dL), BP-lowering medication, lipid-lowering medication. All models were adjusted by follow-up duration (in days).

^1^Model 1 (unregularised): Bayesian logistic regression including all predictors, with no shrinkage priors and no interaction terms.

^2^Model 2 (regularised): Bayesian logistic regression including all predictors with shrinkage priors.

^3^Blockwise regression models (A-I) are regularised Bayesian regressions (using the same priors as Model 2) built using the specific predictors listed in each model. Social factors: age, sex, parental history of CMD, education, financial difficulty, country of birth, residency, site. Behavioural & psychosocial factors: smoking, alcohol, sodium, fibre, physical activity, self-perceived stress, sleep duration. Metabolic factors: SBP, DBP, waist circumference, HDL-C, non-HDL-C, BP-lowering medication, lipid-lowering medication.

^4^Model H excludes education, financial difficulty, country of birth, residency, and site of examination.

^5^Blockwise Model I (full model) are identical to “Model 2” which includes all the predictors (social, behavioural & psychosocial, and metabolic factors).

^6^ΔAUC is the change in AUC relative to reference model (Model A: age-only model).

Based on calibration and discrimination performance, the regularised model (Model 2) was selected for subsequent analyses.

Based on the regularised model (Model 2), we compared predictor blocks against an age-only model as the reference (Model 2 A). Metabolic factors (Model F) gave the largest gain in discrimination (ΔAUC + 0.12 for any cardiometabolic disease and + 0.15 for multimorbidity), while social factors (Model D) added a smaller but meaningful improvement (ΔAUC + 0.08 and + 0.06). Behavioural and psychosocial factors alone (Model E) did not improve discrimination. The full model, including all predictors (Model I), performed best, with AUCs of 0.76 for any cardiometabolic disease and 0.89 for multimorbidity.

We assessed incremental clinical utility by comparing Model H, which excluded socioeconomic and geographical factors, with the full model (Model I). For any cardiometabolic disease, adding socioeconomic and geographical factors showed small but consistent improvements in net benefit across all decision thresholds and in reclassification metrics (NRI 0.066; IDI 0.006). For cardiometabolic multimorbidity, socioeconomic and geographical factors did not improve prediction, with negligible net benefit differences and negative reclassification indices (Supplementary Table A6).

To assess non-additive effects, five interaction terms were added one at a time to the full regularised model (Model 2). Predictive performance was unchanged for both outcomes, with no meaningful improvement in AUC or TPR_FPR10%_ (Supplementary Table A7). We therefore retained the additive model without interactions to derive the risk stratification score.

### Association between predictors and cardiometabolic disease and multimorbidity

**Fig. 1:**
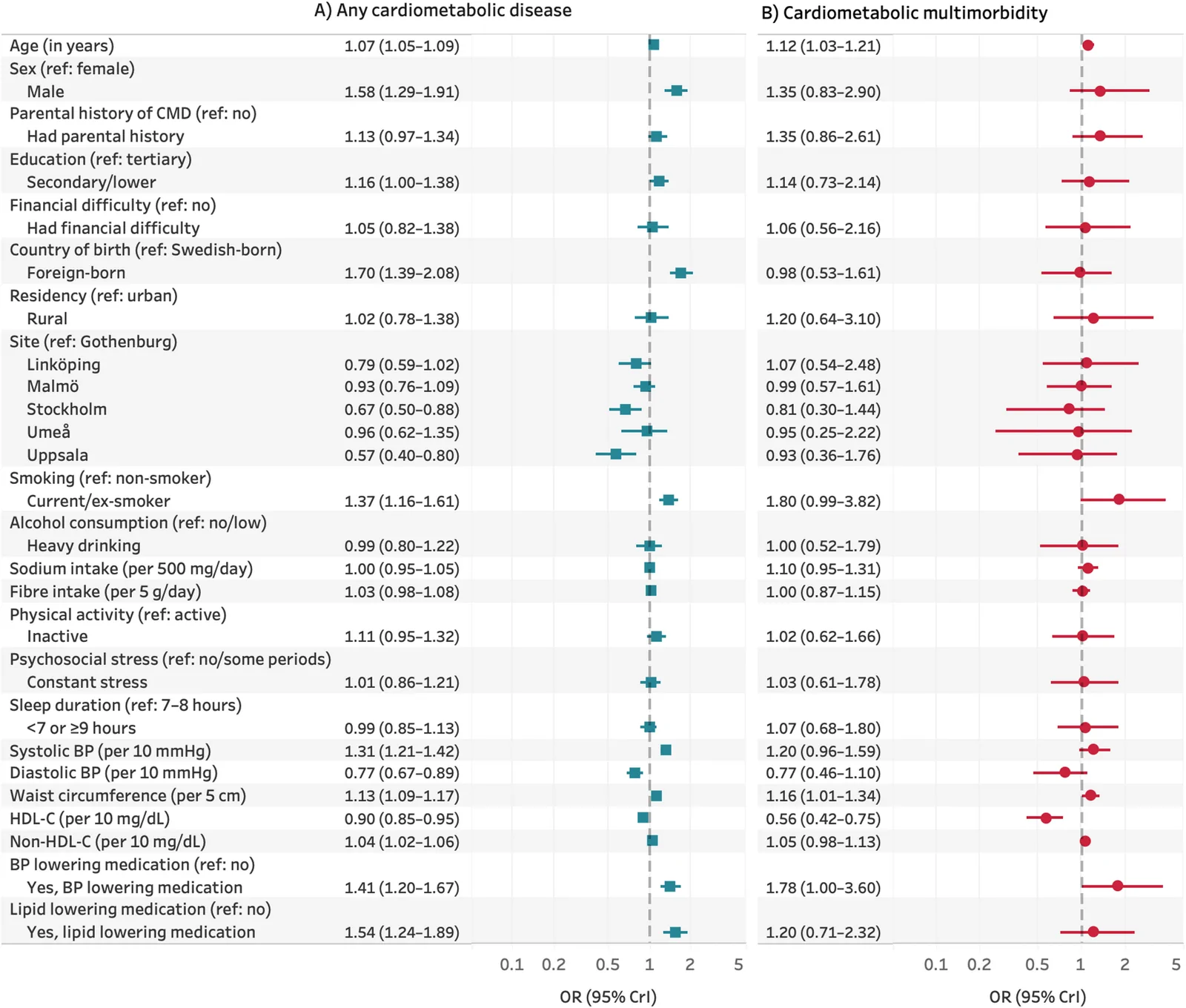
Estimated odds ratios (ORs) from regularised Bayesian logistic regression models for (**A**) any cardiometabolic disease and (**B**) cardiometabolic multimorbidity. OR: odds ratio on logarithmic scale; CMD: cardiometabolic disease; CrI: credible interval. Estimates were calculated based on regularised Bayesian logistic regression, adjusted by follow-up durations. Blue square and red dots represent the median of the posterior distributions, and the line represents 2.5th and 97.5th percentiles of the distributions.

Figure 1 shows the adjusted ORs from the regularised model. For any cardiometabolic disease, odds were higher in foreign-born and male participants and increased with age, smoking, higher systolic blood pressure, and larger waist circumference, while higher HDL-C showed a protective association. For cardiometabolic multimorbidity, estimates were more uncertain. Older age was associated with multimorbidity, and higher HDL-C levels showed a strong protective association. Smoking and blood pressure-lowering medication had elevated point estimates, but CrI included 1.0, indicating uncertainty. Social factors showed limited evidence of association.

### Predicted risk of any cardiometabolic disease and multimorbidity

Predicted risks from the full regularised model were summarised across strata defined by seven key variables: sex, age, country of birth, smoking status, hypertension status, HDL-C, and waist circumference. These variables were chosen based on the magnitude of the estimated ORs and their 95% CrIs, clinical interpretability, and their relevance for representing social and cardiometabolic risk profiles. Continuous variables were categorised using clinically relevant cut-offs to enhance interpretability (Supplementary Table A3).

**Fig. 2:**
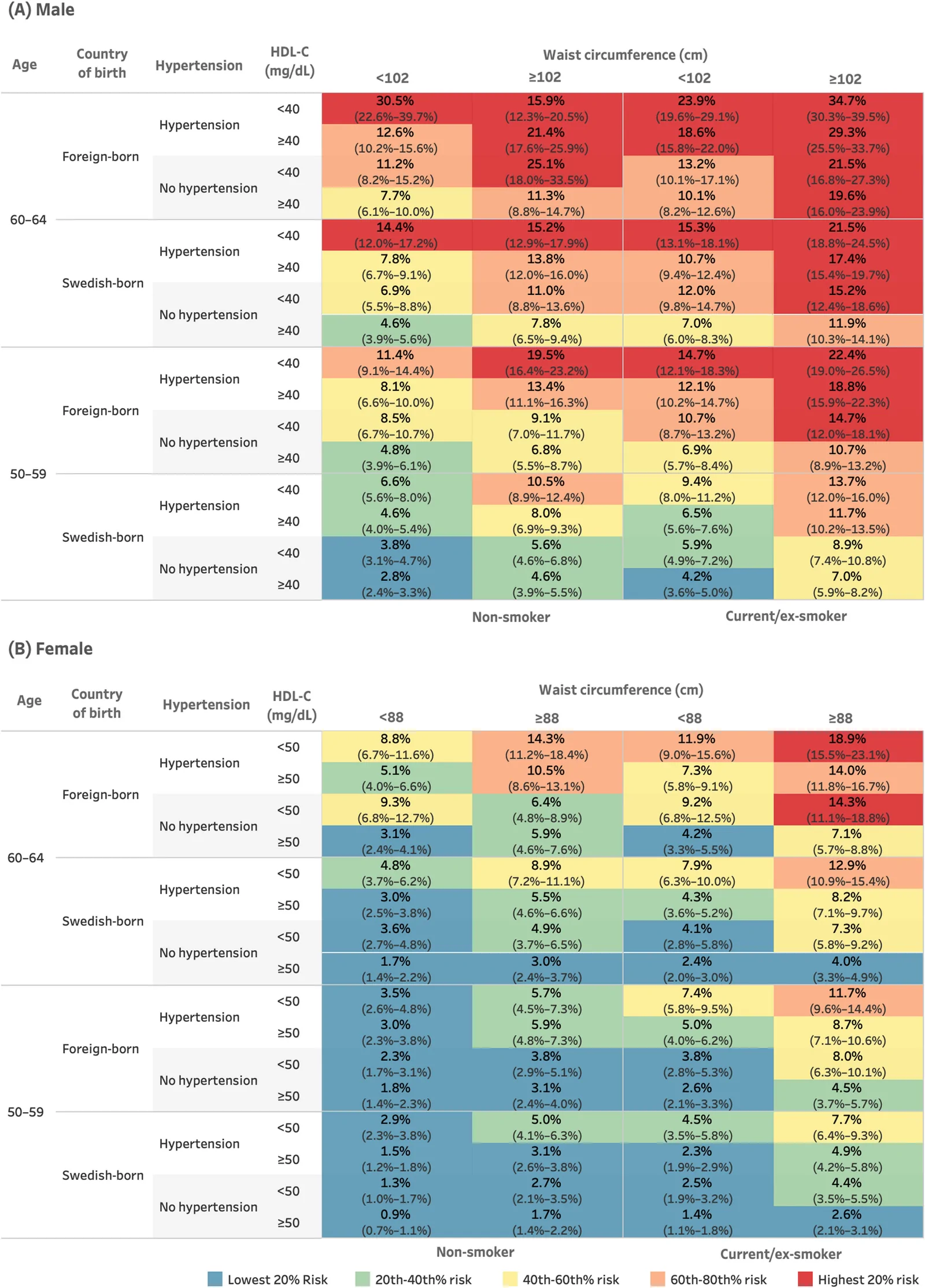
Predicted risk of any cardiometabolic disease based on different combinations of risk profiles. HDL-C: high-density lipoprotein cholesterol. Predicted risk were calculated based on regularised Bayesian logistic regression without interaction (Model 2), including 22 predictors in the models (see Fig. 1). For specific risk profiles, the reported predicted risk represents the median of the posterior distributions from the final regularised Bayesian logistic regression model, and the 95% CrI represents the 2.5th and 97.5th percentiles of the distribution. Hypertension was defined as systolic blood pressure ≥130 mmHg or diastolic blood pressure ≥85 mmHg or took any blood pressure-lowering drugs. Waist circumference and HDL-C were cate-gorised using NCEP-ATP III guideline.

Figure 2 shows the predicted two- to five-year risk of cardiometabolic disease, represented as heatmaps ranging from the lowest 20% risk (blue cells) to the highest 20% risk (red cells). Foreign-born individuals consistently had a higher predicted risk than Swedish-born individuals with similar risk profiles. For example, among non-smoking males aged 60–64 years with hypertension, HDL-C < 40 mg/dL, and waist circumference < 102 cm, predicted risk was 30.5% (95% CrI 22.6%–39.7%) for foreign-born individuals compared to 14.4% (95% CrI 12.0%–17.2%) for Swedish-born individuals. Overall, males had higher predicted risk than females, with the highest risk of 34.7% (95% CrI 30.3%–39.5%) observed among foreign-born current/ex-smokers aged 60–64 years with hypertension, HDL-C < 40 mg/dL, and waist circumference ≥ 102 cm.

**Fig. 3:**
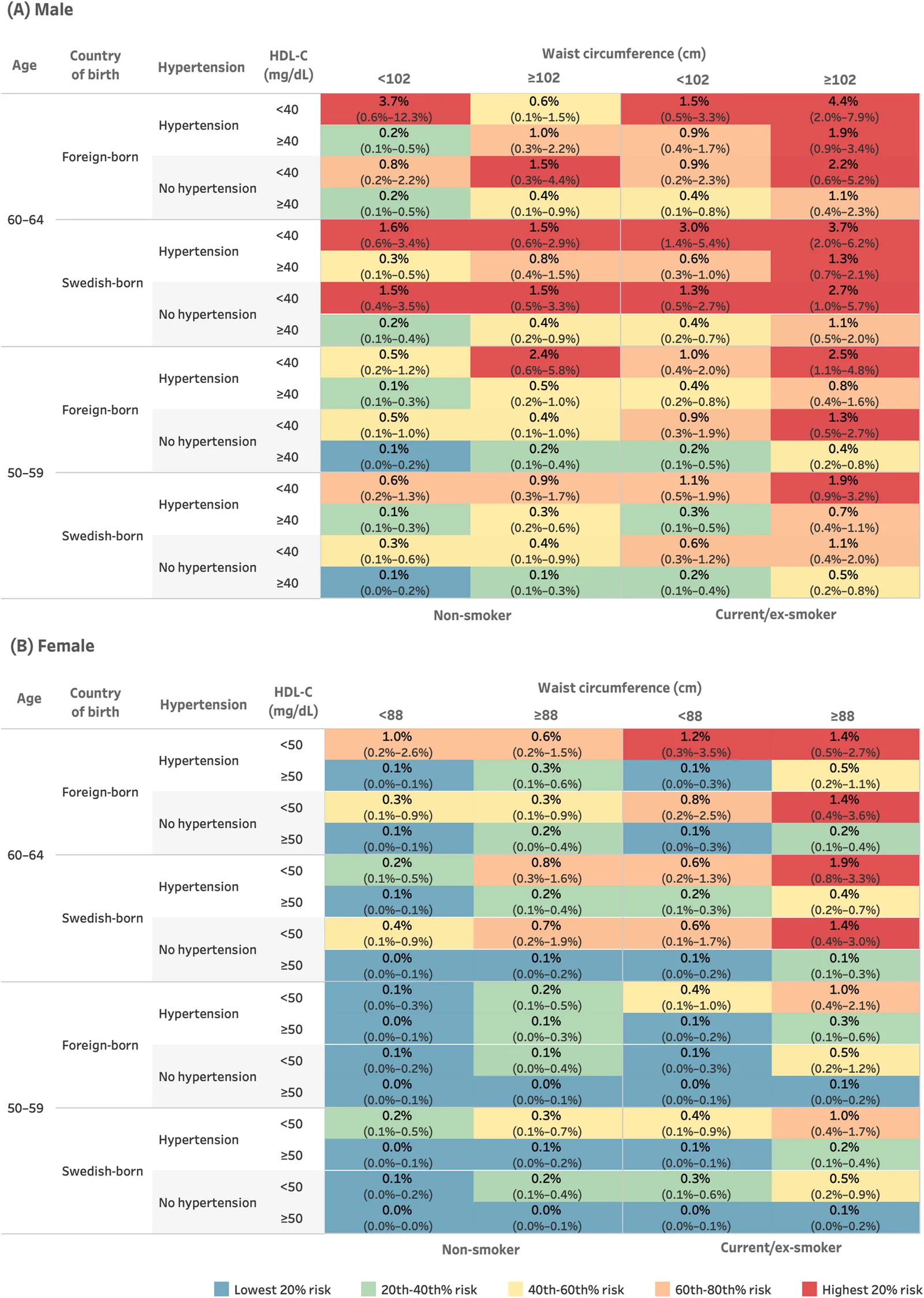
Predicted risk of cardiometabolic multimorbidity based on different combinations of risk profiles. HDL-C: high-density lipoprotein cholesterol. Predicted risk were calculated based on regularised Bayesian logistic regression without interaction (Model 2), including 22 predictors in the models (see Fig. 1). For specific risk profiles, the reported predicted risk represents the median of the posterior distributions from the final regularised Bayesian logistic regression model, and the 95% CrI represents the 2.5th and 97.5th percentiles of the distribution. Hypertension was defined as systolic blood pressure ≥130 mmHg or diastolic blood pressure ≥85 mmHg or took any blood pressure-lowering drugs. Waist circumference and HDL-C were categorised using NCEP-ATP III guideline.

Figure 3 shows the predicted two- to five-year risk of cardiometabolic multimorbidity. Among males, predicted risk ranged from 0.1% (95% CrI 0.0%–0.2%) in the lowest-risk profile to 4.4% (95% CrI 2.0%–7.9%) in the highest-risk profile, while among females it ranged from 0.0% (95% CrI 0.0%–0.0%) to 1.9% (95% CrI 0.8%–3.3%). Differences between foreign-born and Swedish-born individuals with similar risk profiles were smaller for multimorbidity. Low HDL-C and high waist circumference were key contributors to higher multimorbidity risk, with low HDL-C associated with an approximately 1.5–3-fold increase in multimorbidity risk within comparable profiles. Smoking further increased the risk when multiple adverse factors were present.

### Sensitivity analyses

Sensitivity analyses were conducted to examine the influence of missing data, model specification, outcome definition, and comparison with an established cardiovascular risk score. First, Bayesian estimation with multiple imputation showed comparable predictive performance to the complete-case regularised model (Supplementary Table A8, Supplementary Fig. A3). The regularised Bayesian model with multiple imputation yielded AUCs of 0.75 (95% CI 0.73–0.77) for any cardiometabolic disease and 0.89 (95% CI 0.85–0.93) for cardiometabolic multimorbidity, which were similar to those from the complete-case analysis. Second, the BART model did not meaningfully improve discrimination compared with the regularised Bayesian logistic regression model, suggesting that the additive model captured the main predictive patterns in the data (Supplementary Table A8, Supplementary Fig. A3). Third, disease-specific analyses for CVD and T2D showed generally consistent predictor patterns with the main model, although the magnitude of associations differed by outcome (Supplementary Table A9, Supplementary Fig. A4). Male sex was more strongly associated with CVD than with T2D, while waist circumference and HDL-C showed stronger associations with T2D and cardiometabolic multimorbidity. Fourth, the regularised Bayesian logistic regression model showed slightly higher discrimination than SCORE2 for CVD (AUC 0.75 vs. 0.72), any cardiometabolic disease (AUC 0.76 vs. 0.71), and cardiometabolic multimorbidity (AUC 0.89 vs. 0.83) (Supplementary Table A10). However, these comparisons should be interpreted cautiously because SCORE2 was developed to predict 10-year fatal and non-fatal CVD, whereas our model predicts broader cardiometabolic outcomes over 2–5 years.

## Discussion

### Principal findings

Using data from a large population-based prospective cohort in Sweden, we developed and internally validated Bayesian models to predict incident cardiometabolic disease and cardiometabolic multimorbidity using social and cardiometabolic risk factors. Within 2–5 years of follow-up, 5.7% of the participants developed a single cardiometabolic disease and 0.3% developed multimorbidity. Regularised Bayesian logistic regression showed fair discrimination for any cardiometabolic disease (AUC = 0.76) and good discrimination for multimorbidity (AUC = 0.89), with good calibration for both outcomes. However, given the low event count for cardiometabolic multimorbidity, these predictive performances should be interpreted with caution, as they may be unstable or optimistic. Metabolic factors contributed most to discrimination, while behavioural and psychosocial factors contributed minimally. Country of birth was a significant predictor of cardiometabolic disease, consistent with a previous Swedish study^39^, but not for cardiometabolic multimorbidity. Risk stratification matrices visualise how social and clinical factors intersect and associate with the predicted risk of cardiometabolic disease, while also displaying the uncertainty around the estimates.

### Implications for policy and research

Calibration and discrimination provide complementary information on model performance with distinct implications for prevention strategies. For any cardiometabolic disease, good calibration but fair discrimination indicates that while predicted risks aligned with observed incidence, the model had limited ability to isolate a high-risk subgroup (low TPR)^32^. Consequently, targeted screening alone would likely miss many future cases^40,41^, supporting the use of population-wide primary prevention alongside high-risk strategies^32,42^. For cardiometabolic multimorbidity, discrimination was higher, although this finding should be interpreted cautiously, given the low number of events. The risk stratification matrices may therefore be useful as exploratory tools for visualising risk patterns across social and clinical risk factors, rather than as tools for immediate clinical decision-making.

Disadvantaged social conditions may contribute to cumulative risk through chronic stress pathways, inflammation, and structural barriers to healthcare access^18,43^. Incorporating social factors into risk prediction is therefore important, as excluding them may overlook socially patterned differences in risk and prevention needs^10^. In our study, the risk stratification matrices showed visible differences in predicted risk of any cardiometabolic disease between Swedish-born and foreign-born participants with otherwise similar risk profiles, but these differences were less visible for cardiometabolic multimorbidity. While the incremental value of socioeconomic and geographical factors was modest for any cardiometabolic disease and negligible for multimorbidity, these variables may still provide additional information on risk patterns and equity-related differences. Whether incorporating these variables improves clinical decision-making or patient outcomes remains to be established.

A previous study in Stockholm using SCORE2 illustrates the challenge of assessing country of birth only after a risk score has been calculated^44^. The study found that foreign-born individuals had lower 10-year CVD risk, but country of birth was treated solely as a stratifying variable rather than being integrated into the risk score. This approach can describe differences in predicted risk between groups, but it does not allow migration-related or social factors to contribute to individual risk prediction. The association also attenuated after adjustment for alcohol, likely reflecting higher consumption among Swedish-born individuals^44^. This also illustrates that standard cardiovascular risk prediction models may not fully capture social gradients in cardiometabolic risk.

Country of birth should be interpreted as a context-specific marker rather than a fixed individual risk factor. In Sweden, foreign-born status may partly capture migration history, region of origin, early-life exposures, socioeconomic position, language and healthcare access, discrimination, and behavioural or contextual factors^45,46^. If the score is applied outside Sweden, this variable should not be transferred directly but should be redefined using locally relevant social determinants^17,18^ and structural barriers to healthcare, followed by external validation in the target population.

Furthermore, the Bayesian framework provides credible intervals around individual predicted risks, which support more transparent risk communication than conventional point estimates alone^21,29^. Credible intervals have a direct probabilistic interpretation, representing the range of plausible predicted risks given the observed data and model assumptions. This differs from conventional confidence intervals, which cannot be directly interpreted as the probability that the specific interval contains the true value. Presenting credible intervals alongside predicted risks can therefore make uncertainty more explicit, helping clinicians distinguish relatively precise estimates from those that are more uncertain or close to possible decision thresholds.

### Strengths and limitations

The strength of this study is the use of a Bayesian framework for prognostic risk prediction, which moves beyond single-point estimates by providing posterior distributions for individual risk and enabling uncertainty quantification to support clinical interpretation and shared decision making^21^. Another strength is the inclusion of social, behavioural, psychosocial, and metabolic predictors, enabling a more holistic risk assessment than models restricted to traditional clinical variables. In addition, the risk stratification matrices add value by offering an interpretable visual summary of predicted risk across different combinations of risk profiles, without the need for complex computational tools at the point of care^9,12,13^.

Several limitations should be acknowledged. *First*, the relatively short follow-up may underestimate longer-term risk and affect model stability, particularly for multimorbidity, given the low event rate (0.3%), which may capture mainly early onset or more aggressive disease transitions. Future studies should validate these findings over longer periods, such as 10 years or longer, to assess long-term performance and more common progression patterns. *Second*, the low number of multimorbidity events limited our ability to model disease sequences or time intervals between diagnoses, which would have helped us better understand the sequential ordering and progression of cardiometabolic diseases. The low event count may also have affected the stability of the multimorbidity model and led to optimistic performance estimates, particularly for discrimination. *Third*, the outcome of any cardiometabolic disease includes T2D, heart disease (including heart failure), and stroke. Although disease-specific sensitivity analyses showed generally consistent predictor patterns, these conditions are not identical, and some associations differed in magnitude across outcomes. Therefore, the model should be interpreted as predicting overall cardiometabolic disease risk rather than disease-specific risk for any single condition. *Fourth*, although all the models adjusted for follow-up duration, logistic regression does not fully account for differences in time-to-event. Survival-based models may be more appropriate when longer follow-up and more events become available. *Fifth*, the findings are based on Swedish adults aged 50–64 years and may not be generalisable to other age groups or settings without external validation, for example, using another Swedish database. *Sixth*, foreign-born status is heterogeneous and may mask subgroup differences by region of origin or duration of residence, which should be examined in future work. Future work should aim to disaggregate this, for example, by length of stay in Sweden, or country of origin, to better capture the complex and intersecting pathways linking migration and cardiometabolic health. *Seventh*, several predictors were self-reported and may be affected by recall or social desirability bias, and risk factors were measured at baseline without accounting for changes during follow-up. *Finally*, SCAPIS does not provide weighting variables that would allow for weighted analyses. However, previous reports indicate that the risk factor distributions are similar to the target population, which may help minimise concerns about the validity of the findings^47^.

## Conclusion

Our regularised Bayesian logistic regression model showed fair discrimination for any cardiometabolic disease and higher discrimination for multimorbidity, with good calibration. The multimorbidity findings should be interpreted cautiously, given the low number of events. Social determinants added modest incremental information for any cardiometabolic disease, while credible intervals may support more transparent communication of individual risk uncertainty. Further external validation, calibration assessment, and evaluation of its usefulness for clinical decision-making are needed before implementation in clinical practice.

## Supplementary Information

Below is the link to the electronic supplementary material.

Supplementary Material 1

Supplementary Material 2

## Data Availability

The data underlying this study cannot be shared publicly due to the privacy of individuals who participated in the study. However, all data used in this study are available from SCAPIS (www.scapis.org) for researcher with an application. The study was registered with SCAPIS and received approval for data access (Petition-447). R codes are available in https://github.com/kanindya/cmdpredict_bayesian.

## Declarations

### Patient and public involvement

This study used pseudonymised national data and thus, patients and/or the public were not involved in the design, conduct, reporting, or dissemination planning of the research.
