# Supplementary material for: Integrating social and cardiometabolic risk factors in predicting cardiometabolic multimorbidity: a Bayesian model development and internal validation in Swedish middle-aged adults
Source: Sci Rep. 2026 Aug 25;16:26679. doi: 10.1038/s41598-026-67901-3 (PMC13507255; doi:10.1038/s41598-026-67901-3)
Supplement: Supplementary file 2 [file 41598_2026_67901_MOESM2_ESM.docx]

**SUPPLEMENTARY MATERIALS**

Table A1***.*** Percentages of missing data

| **Variables** | N | % |
| --- | --- | --- |
| **All SCAPIS participants** | **30154** |  |
| Exclusion criteria (see Figure A1) | 15671 |  |
| **Total eligible sample (for sensitivity analysis with imputation)** | **14483** | **100** |
| Participants with missing value | 2519 | 17.4 |
| **Total sample for main analysis** | **11964** | **82.6** |
| **Variables with missing value:** |  |  |
| Physical activity | 1097 | 7.6 |
| Sleep duration | 613 | 4.2 |
| Family history of CVD | 605 | 4.2 |
| Psychosocial stress | 599 | 4.1 |
| Financial difficulty | 517 | 3.6 |
| Alcohol consumption | 493 | 3.4 |
| Smoking | 471 | 3.3 |
| Education | 452 | 3.1 |
| Country of birth | 424 | 2.9 |
| Sodium intake | 364 | 2.5 |
| Fibrer intake | 364 | 2.5 |
| Diastolic blood pressure | 70 | 0.5 |
| Systolic blood pressure | 70 | 0.5 |
| Residency (DeSO) | 68 | 0.5 |
| Non-HDL-C | 48 | 0.3 |
| HDL-C | 46 | 0.3 |
| Waist circumference | 5 | 0.0 |
| Age | 0 | 0.0 |
| Blood pressure medication | 0 | 0.0 |
| Lipid lowering medication | 0 | 0.0 |
| Sex | 0 | 0.0 |
| Site | 0 | 0.0 |

**Notes**: Sample flowchart is available in Figure A1.

Table A2***.*** List of variables

| **Variables** | **Original variables: description** | **Variables’ measurement** |
| --- | --- | --- |
| **Outcomes** | | |
| Cardiometabolic disease | The assessment of cardiometabolic disease was based on multiple resources, including: 1) received diagnosis or coronary revascularization based on the NPR after the interview; 3) received glucose-lowering medications in the SPDR after the interview.  Type 2 diabetes:  ICD-10 Code:   - E11–14   ATC Code:   - A10A (for those without Type 1 diabetes), A10B   Heart disease:  ICD-10 Code:   - Coronary heart disease: I20–I25 - Heart failure: I50 - Coronary revascularization: FNA, FNB, FNC, FND, FNE, FNG   Stroke:  ICD-10 Code:   - Ischemic stroke: I63 - Hemorrhagic stroke: I60, I61 - Not specified: I64 | Type 2 diabetes: 0: No  1: Yes (had the condition based on register data)  Heart disease:  0: No  1: Yes (had the condition based on register data)  Stroke:  0: No  1: Yes (had the condition based on register data)  Number of cardiometabolic disease:  0: No CMD  1: Single CMD (had 1 cardiometabolic disease)  2: Cardiometabolic multimorbidity (had ≥2 cardiometabolic diseases)  Any cardiometabolic disease:  0: No CMD  1: Any CMD (Had at least 1 cardiometabolic disease)  Cardiometabolic multimorbidity:  0: No/single CMD  1: Cardiometabolic CMD (at least 2 cardiometabolic diseases) |
| **Cardiometabolic risk factors (independent variables)** | | |
| Age | AgeAtVisitOne: Age at study visit 1, rounded to 1 decimal | Measured in years |
| Sex | Sex | 0: Female  1: Male |
| Family history of cardiometabolic disease | cqdf002: Family history of diabetes, subject’s mother  cqdf003: Family history of diabetes, subject’s father  cqdf017: Family history of myocardial infarction, subject’s mother  cqdf018: Family history of myocardial infarction, subject’s father  cqdf022: Family history of stroke, subject’s father  cqdf023: Family history of stroke, subject’s mother | 0: No (if respondents answered “no” for family history of diabetes, myocardial infarction, and stroke)  1: Yes (if respondents had family history of diabetes, myocardial infarction and stroke from mother and/or father) |
| Educational attainment | cqed001: Highest completed level of education | 0: Tertiary (university/college)  1: Secondary (upper secondary, higher secondary, vocational or equivalen) or lower (did not complete primary or other compulsory education, lower secondary or equivalent (maximum 9 years)) |
| Financial difficulty | cqed018: Difficulties managing regular expenses, last 12 months | 0: No  1: Yes |
| Country of birth | cqli009: Born in Sweden | 0: Sweden (Swedish-born)  1: Outside Sweden (Foreign-bron) |
| Type of residence | DeSO classification | 0: Urban (area B, C)  1: Rural (area A) |
| Site | Site: Site of examination | 0: Gothenburg  1: Linköping  2: Malmö  3: Stockholm  4: Umeå  5: Uppsala |
| Smoking | derived_smoke_status: Derived variable based on current smoking status and information on smoking status at biological sampling. | 0: Non-smoker  1: Current smoker or ex-smoker |
| Alcohol consumption | **cqah001**: Frequency having an alcoholic drink, last year: (0) Never; (1) Once a month; (2) 2–4 times/month; (3) 2–3 times/week; (4) ≥ 4 times per week  **cqah002**: Number of alcoholic drinks on a typical drinking day year: (0) 1–2; (1) 3–4; (2) 5–6; (3) 7–9; (4) ≥10  **cqah003**: Frequency having >6 alcoholic drinks on one occasion: 0) Never; (1) <1 per month; (2) Every month; (3) Every week; (4) Daily/almost everyday  **cqah004**: Frequency not being able to stop drinking, last year: 0) Never; (1) <1 per month; (2) Every month; (3) Every week; (4) Daily/almost everyday  **cqah005**: Frequency failing to do what is expected due to drinking, last year: 0) Never; (1) <1 per month; (2) Every month; (3) Every week; (4) Daily/almost everyday  **cqah006**: Frequency needing a drink in the morning after drinking the night before, last year: 0) Never; (1) <1 per month; (2) Every month; (3) Every week; (4) Daily/almost everyday  **cqah007**: Frequency feeling guilty about drinking, last year: 0) Never; (1) <1 per month; (2) Every month; (3) Every week; (4) Daily/almost everyday  **cqah008**: Frequency drinking resulting in blackouts, last year: 0) Never; (1) <1 per month; (2) Every month; (3) Every week; (4) Daily/almost everyday  **cqah009**: Injuries caused by drinking: 0) No; (4) Yes  **cqah010**: Concerns expressed by others about  drinking: 0) No; (4) Yes | Alcohol Use Disorders Identification (AUDIT) scoring.  Each question is scored from 0 to 4, with the total score range from 0 to 40. SCAPIS slightly modified the last two questions (cqah009 and cqah010) by using binary options (No/Yes) instead of the original three categories (No/ Yes, but not in the last year/ Yes, during the last year). For these two questions, “No” was coded as 0, and “Yes” was coded as 4.  0: Abstinence/low-risk (score<8)  1: Hazardous alcohol consumption/alcohol dependence (score≥8) |
| Sodium intake | natrium: calculated nutrition value, sodium (milligram/mg) | Converted to gram |
| Fiber intake | fibre: calculated nutrition value, sodium (milligram/mg) | Converted to gram |
| Physical activity | validdays: number of valid days of accelerometer recording  weartimedaily: Accelerometer wear time (minutes per day)  mpa: Moderate intensity physical activity (MPA), total minutes  vpa: Vigorous-intensity physical activity (VPA), total minutes  seddaily: Sedentary (SED), average minutes per day | WHO guidelines of physical activity: (low VPA <75 min/week and low MPA<150 min/week). High daily sedentary was defined as ≥9.5 hours/day.  For participants with less than 7 days of valid data (validdays<7), the daily threshold used was 10.7 for 75 min/week, 21.4 for 150 min/week, and 42.8 min for 300 min/week.  0: Low risk  1: At-risk (highly sedentary [≥9.5 hours/day] AND low VPA [<75 min/week]) |
| Psychosocial stress | cqsle001: By stress we mean feeling tense, irritable, anxious or having sleeping difficulties as a result of conditions at work or at home. Did you experience this? | Adaptation from Rosengen, et al. (2004).  0: No/several period (Never experienced stress/have experienced a period of stress/some stressful period in the last five years)  1: Constant stress (Consistent stress in the past year/constant stress in the last five years) |
| Sleep duration | cqsh002: Hours of sleep per night under usual  circumstances | Adaptation from St-Onge et al. (2016) and AHA Life’s Simple 7 (2022).  0: Sufficient (≥7 hours)  1: Insufficient/prolonged (<7 hours or ≥ 9 hours) |
| Elevated waist circumference | Waist: Waist circumference (cm) | Measured in cm |
| HDL-C | HdlFormattedResult: HDL Numerical result – national variable (mmol/L) | Converted from mmol/L to mg/dL |
| Non-HDL-C | HdlFormattedResult: HDL Numerical result – national variable (mmol/L)  CholesterolFormattedResult: Cholesterol Numerical result (unit: mmol/L) | Non-HDL-C = Total cholesterol – HDL-C  Converted from mmol/L to mg/dL |
| Systolic blood pressure | SBP_Mean: Systolic blood pressure, mean brachial (recommended for general analysis, mmHg) | Measured in mmHg |
| Diastolic blood pressure | DBP_Mean: Diastolic blood pressure, mean brachial (recommended for general analysis, mmHg) | Measured in mmHg |
| Blood pressure lowering medication | Prescribed drug register, ATC codes: C02, C03,  C07, C08, C09, excluding C02AC02 and C07AA07*  *Reference: National Board of Health and Welfare. Statistics on pharmaceuticals 2022. 2023. Available at: <https://www>.socialstyrelsen.se/globalassets/sharepoint-dokument/artikelkatalog/xceptic/2023-3-8457.pdf | 0: No  1: Yes (had been prescribed by blood pressure lowering drugs before the interview) |
| Lipid lowering medication | Prescribed drug register, ATC codes: C10  *Reference: National Board of Health and Welfare. Statistics on pharmaceuticals 2022. 2023. Available at: <https://www>.socialstyrelsen.se/globalassets/sharepoint-dokument/artikelkatalog/xceptic/2023-3-8457.pdf | 0: No  1: Yes (had been prescribed by lipid lowering drugs before the interview) |

Table A3*.* Categorisation for continues variables in risk prediction matrix

| **Variables** | **Binary variables** |
| --- | --- |
| Age | 0: 50–59 years  1: 60–64 years |
| Hypertension | 0: No  1: Yes (systolic BP ≥ 130 or diastolic BP ≥ 85 mmHg or had been prescribed by antihypertensive drugs)  The Third Report of the National Cholesterol Education Program Expert Panel on Detection, Evaluation, and Treatment of High Blood Cholesterol in Adults (NCEP ATP III). |
| Waist circumference | 0: Normal (<102 cm for males & <88 cm for females)  1: High (≥102 cm for males & ≥88 cm for females)  The Third Report of the National Cholesterol Education Program Expert Panel on Detection, Evaluation, and Treatment of High Blood Cholesterol in Adults (NCEP ATP III). |
| HDL-C | 0: Normal (≥40 mg/dL in males and ≥50 mg/dL in females)  1: Low (<40 mg/dL in males and <50 mg/dL in females)  The Third Report of the National Cholesterol Education Program Expert Panel on Detection, Evaluation, and Treatment of High Blood Cholesterol in Adults (NCEP ATP III). |

Table A4. Disease sequence among participants who developed incident cardiometabolic disease in the complete-case analytical sample

| **Disease sequence** | **n** | **%** |
| --- | --- | --- |
| **No cardiometabolic disease** | 11,242 | 93.97 |
| **Single condition** |  |  |
| D (T2D only) | 229 | 1.91 |
| H (heart disease only) | 385 | 3.22 |
| S (stroke only) | 68 | 0.57 |
| **Two conditions — sequential** |  |  |
| D → H | 11 | 0.09 |
| H → D | 21 | 0.18 |
| D → S | 1 | 0.01 |
| S → D | 2 | 0.02 |
| H → S | 2 | 0.02 |
| **Two conditions — simultaneous** |  |  |
| D + H | 2 | 0.02 |
| **Three conditions** |  |  |
| H → (D + S) | 1 | 0.01 |
| Total | 11,964 | 100 |

**Notes:** D = type 2 diabetes; H = heart disease (ischaemic heart disease or heart failure); S = stroke. Arrow (→) indicates sequential diagnosis, plus sign (+) indicates simultaneous diagnosis on the same date.

Table A5***.*** Time between first and second diagnosis among participants with cardiometabolic multimorbidity (n=40)

| **Disease sequence** | **Days between first and second diagnosis** | | | | |
| --- | --- | --- | --- | --- | --- |
|  | Within 1 month | 1–3 months | 3–12 months | >1 year | Total |
| D → H | 1 (9.1%) | 2 (18.2%) | 5 (45.5%) | 3 (27.3%) | 11 (100.0%) |
| H → D | 2 (9.5%) | 0 (0.0%) | 4 (19.1%) | 15 (71.4%) | 21 (100.0%) |
| D → S | 0 (0.0%) | 0 (0.0%) | 1 (100.0%) | 0 (0.0%) | 1 (100.0%) |
| S → D | 0 (0.0%) | 1 (50.0%) | 0 (0.0%) | 1 (50.0%) | 2 (100.0%) |
| H → S | 0 (0.0%) | 1 (50.0%) | 0 (0.0%) | 1 (50.0%) | 2 (100.0%) |
| D + H | 2 (100.0%) | 0 (0.0%) | 0 (0.0%) | 0 (0.0%) | 2 (100.0%) |
| H → (D + S) | 1 (100.0%) | 0 (0.0%) | 0 (0.0%) | 0 (0.0%) | 1 (100.0%) |
| Total | 6 (15.0%) | 4 (10.0%) | 10 (25.0%) | 20 (50.0%) | 40 (100.0%) |

**Notes:** D = type 2 diabetes; H = heart disease (ischaemic heart disease or heart failure); S = stroke. Arrow (→) indicates sequential diagnosis, plus sign (+) indicates simultaneous diagnosis on the same date.

Table A6***.*** Incremental clinical utility of socioeconomic and geographical factors: Model H (all predictors except socioeconomic and geographical factors) vs Model I (full model) for cardiometabolic disease and multimorbidity

|  | **Model H:** Age, sex, family history, behavioural, psychosocial, and metabolic factors^1^ | **Model I:** Full model (all predictors)^2^ | **Increment** (Model I vs Model H) |
| --- | --- | --- | --- |
| **Any cardiometabolic disease (n=11,964; n events=722)** | |  |  |
| **Discrimination** |  |  |  |
| AUC (95% CI) | 0.74 (0.73–0.77) | 0.76 (0.74–0.78) | 0.02 |
| TPR_FPR 10%_ | 0.35 | 0.38 | 0.025 |
| **Net benefit (Decision Curve Analysis)^3^** | |  |  |
| At 5% threshold | 0.0264 | 0.0275 | +0.0011 |
| At 10% threshold | 0.0127 | 0.0134 | +0.0007 |
| At 15% threshold | 0.0054 | 0.0071 | +0.0017 |
| **Reclassification** |  |  |  |
| NRI (95% CI) | — | — | 0.066 (0.031–0.101) |
| IDI (95% CI) | — | — | 0.006 (0.003–0.008) |
| **Cardiometabolic multimorbidity (n=11,964; n events=40)** | |  |  |
| **Discrimination** |  |  |  |
| AUC (95% CI) | 0.89 (0.86–0.92) | 0.89 (0.85–0.93) | −0.007 |
| TPR_FPR 10%_ | 0.75 | 0.75 | 0 |
| **Net benefit (Decision Curve Analysis)^3^** | |  |  |
| At 0.5% threshold | 0.0018 | 0.0019 | +0.0001 |
| At 2% threshold | 0.0004 | 0.0005 | +0.0001 |
| At 3% threshold | 0.0002 | 0.0001 | −0.0001 |
| **Reclassification** |  |  |  |
| NRI (95% CI) | — | — | −0.067 (−0.204–0.055) |
| IDI (95% CI) | — | — | −0.002 (−0.004–0.000) |

**Notes:** AUC: area under the receiver operating characteristic curve estimated from 10-fold cross-validation; TPR: true positive rate; FPR: false positive rate; NRI: net reclassification improvement using risk categories 5%, 10%, and 15%; IDI: integrated discrimination improvement.

1 Model H includes age, sex, family history of cardiometabolic disease, behavioural, psychosocial, and metabolic factors.
2 Model I (full model) additionally includes education, financial difficulty, country of birth, residency, and site of examination. Net benefit values represent the number of additional true cases correctly identified per patient screened minus the weighted false positives at each decision threshold.
3 Net benefit values represent the number of additional true cases correctly identified per patient screened minus the weighted false positives at each decision threshold. Decision curve analysis for multimorbidity uses lower thresholds (0.5%–3%) reflecting the low event rate (0.3%); a 5% threshold exceeds the predicted probability range for this outcome and is therefore not reported.

Table A7***.*** Predictive performance of regularised Bayesian models including interaction for any cardiometabolic disease and multimorbidity

| **Models** | **AUC (95% CI)** | **TPR_FPR 10%_** |
| --- | --- | --- |
| **Any cardiometabolic disease (n=11,964; n events=722)** |  |  |
| *Interaction models^1^* |  |  |
| (A) Age × sex | 0.76 (0.74–0.78) | 0.38 |
| (B) Country of birth × education | 0.76 (0.73–0.78) | 0.37 |
| (C) Sex × HDL-C | 0.76 (0.74–0.78) | 0.38 |
| (D) Smoking × non-HDL-C | 0.76 (0.74–0.78) | 0.38 |
| (E) Waist circumference × non-HDL-C | 0.76 (0.73–0.79) | 0.37 |
| **Cardiometabolic multimorbidity (n=11,964; n events=40)** |  |  |
| *Interaction models^1^* |  |  |
| (A) Age × sex | 0.89 (0.85–0.93) | 0.73 |
| (B) Country of birth × education | 0.89 (0.85–0.93) | 0.73 |
| (C) Sex × HDL-C | 0.89 (0.86–0.93) | 0.73 |
| (D) Smoking × non-HDL-C | 0.90 (0.86–0.93) | 0.70 |
| (E) Waist circumference × non-HDL-C | 0.89 (0.86–0.93) | 0.73 |

**Notes**: AUC: area under the receiver operating curve (estimated based on 10-fold cross-validation); CI: confidence interval; TPR: true positive rate; FPR: false positive rate**;** TPR_FPR10%_: true positive rate at a 10% false positive rate. The addition of interaction terms did not meaningfully improve model discrimination (AUCs were mostly unchanged when rounded to two decimal places).

1 Interaction models (A-E) are similar to Model 2 (regularised Bayesian logistic regression), but with the addition of the single interaction term indicated (e.g., Model A = age × sex + other predictors). List of predictors: age (in years), sex, family history of cardiometabolic disease, educational attainment, financial difficulty, country of birth, residency, site of examination, smoking, alcohol consumption, sodium intake (gram/day), fibre intake (gram/day), physical activity, self-perceived stress, sleep duration, SBP (mmHg), DBP (mmHg), waist circumference (cm), HDL-C (mg/dL), non-HDL-C (mg/dL), BP-lowering medication, lipid-lowering medication. All models were adjusted by follow-up duration (in days).

Table A8***.*** Predictive performance for any cardiometabolic disease and multimorbidity based on regularised Bayesian with multiple imputation and Bayesian Additive Tree Regression

| **Models** | **AUC (95% CI)** | **TPR_FPR 10%_** |
| --- | --- | --- |
| **Any cardiometabolic disease** |  |  |
| Regularised Bayesian logistic regression *with* complete case analysis | 0.76 (0.74–0.78) | 0.38 |
| Regularised Bayesian logistic regression *with* multiple imputation | 0.75 (0.73–0.77) | 0.39 |
| Bayesian additive regression trees (BART) | 0.75 (0.73–0.77) | 0.37 |
| **Cardiometabolic multimorbidity** |  |  |
| Regularised Bayesian logistic regression *with* complete case analysis | 0.89 (0.85–0.93) | 0.75 |
| Regularised Bayesian logistic regression *with* multiple imputation | 0.89 (0.86–0.93) | 0.73 |
| Bayesian additive regression trees (BART) | 0.84 (0.78–0.89) | 0.63 |

**Notes**: AUC: area under the receiver operating curve (estimated based on 10-fold cross-validation); CI: confidence interval; TPR: true positive rate; FPR: false positive rate**;** TPR_FPR10%_: true positive rate at a 10% false positive rate

Table A9***.*** Estimated odds ratios (ORs) from regularised Bayesian logistic regression models for any cardiometabolic disease, cardiometabolic multimorbidity, heart disease and stroke, and diabetes

| Variables | Any CMD | Multimorbidity | Heart disease/stroke | Diabetes |
| --- | --- | --- | --- | --- |
|  | AOR (95% CI) | AOR (95% CI) | AOR (95% CI) | AOR (95% CI) |
| Age (in years) | 1.00 (1.00–1.00) | 1.12 (1.03–1.21) | 1.08 (1.05–1.10) | 1.05 (1.02–1.09) |
| Sex (ref: female) | 1.07 (1.05–1.09) |  |  |  |
| Male | 1.58 (1.29–1.91) | 1.35 (0.83–2.90) | 2.43 (1.92–3.05) | 1.30 (1.01–1.74) |
| Parental history of CMD (ref: no) |  |  |  |  |
| Had parental history | 1.13 (0.97–1.34) | 1.35 (0.86–2.61) | 1.08 (0.91–1.29) | 1.25 (1.00–1.59) |
| Education (ref: tertiary) |  |  |  |  |
| Secondary/lower | 1.16 (1.00–1.38) | 1.14 (0.73–2.14) | 1.11 (0.94–1.36) | 1.14 (0.92–1.48) |
| Financial difficulty (ref: no) |  |  |  |  |
| Had financial difficulty | 1.05 (0.82–1.38) | 1.06 (0.56–2.16) | 1.11 (0.84–1.54) | 1.01 (0.72–1.38) |
| Country of birth (ref: Swedish-born) |  |  |  |  |
| Foreign-born | 1.70 (1.39–2.08) | 0.98 (0.53–1.61) | 1.37 (1.09–1.70) | 1.90 (1.43–2.47) |
| Residency (ref: urban) |  |  |  |  |
| Rural | 1.02 (0.78–1.38) | 1.20 (0.64–3.10) | 1.02 (0.75–1.42) | 1.06 (0.77–1.58) |
| Site (ref: Gothenburg) |  |  |  |  |
| Linköping | 0.79 (0.59–1.02) | 1.07 (0.54–2.48) | 0.70 (0.50–0.98) | 0.96 (0.67–1.31) |
| Malmö | 0.93 (0.76–1.09) | 0.99 (0.57–1.61) | 0.85 (0.67–1.05) | 1.05 (0.86–1.33) |
| Stockholm | 0.67 (0.50–0.88) | 0.81 (0.30–1.44) | 0.69 (0.51–0.94) | 0.74 (0.49–1.03) |
| Umeå | 0.96 (0.62–1.35) | 0.95 (0.25–2.22) | 0.87 (0.46–1.29) | 1.03 (0.62–1.80) |
| Uppsala | 0.57 (0.40–0.80) | 0.93 (0.36–1.76) | 0.47 (0.30–0.70) | 0.99 (0.70–1.36) |
| Smoking (ref: non-smoker) |  |  |  |  |
| Current/ex-smoker | 1.37 (1.16–1.61) | 1.80 (0.99–3.82) | 1.39 (1.16–1.67) | 1.24 (0.99–1.58) |
| Alcohol consumption (ref: no/low) |  |  |  |  |
| Heavy drinking | 0.99 (0.80–1.22) | 1.00 (0.52–1.79) | 1.06 (0.86–1.33) | 0.93 (0.65–1.21) |
| Sodium intake (per 500 mg/day) | 1.00 (0.95–1.05) | 1.10 (0.95–1.31) | 1.02 (0.96–1.08) | 1.02 (0.96–1.10) |
| Fibre intake (per 5 g/day) | 1.03 (0.98–1.08) | 1.00 (0.87–1.15) | 1.01 (0.96–1.07) | 1.01 (0.95–1.07) |
| Physical activity (ref: active) |  |  |  |  |
| Inactive | 1.11 (0.95–1.32) | 1.02 (0.62–1.66) | 1.05 (0.89–1.26) | 1.10 (0.89–1.41) |
| Psychosocial stress (ref: no/some periods) |  |  |  |  |
| Constant stress | 1.01 (0.86–1.21) | 1.03 (0.61–1.78) | 0.93 (0.74–1.13) | 1.17 (0.94–1.51) |
| Sleep duration (ref: 7–8 hours) |  |  |  |  |
| <7 or ≥9 hours | 0.99 (0.85–1.13) | 1.07 (0.68–1.80) | 0.96 (0.81–1.13) | 1.01 (0.83–1.25) |
| Systolic BP (per 10 mmHg) | 1.31 (1.21–1.42) | 1.20 (0.96–1.59) | 1.27 (1.16–1.39) | 1.28 (1.13–1.44) |
| Diastolic BP (per 10 mmHg) | 0.77 (0.67–0.89) | 0.77 (0.46–1.10) | 0.83 (0.71–0.97) | 0.77 (0.63–0.94) |
| Waist circumference (per 5 cm) | 1.13 (1.09–1.17) | 1.16 (1.01–1.34) | 1.02 (0.97–1.06) | 1.28 (1.21–1.35) |
| HDL-C (per 10 mg/dL) | 0.90 (0.85–0.95) | 0.56 (0.42–0.75) | 0.96 (0.90–1.02) | 0.74 (0.67–0.81) |
| Non-HDL-C (per 10 mg/dL) | 1.04 (1.02–1.06) | 1.05 (0.98–1.13) | 1.05 (1.03–1.08) | 1.01 (0.98–1.03) |
| BP lowering medication (ref: no) |  |  |  |  |
| Yes, BP lowering medication | 1.41 (1.20–1.67) | 1.78 (1.00–3.60) | 1.58 (1.30–1.91) | 1.19 (0.96–1.54) |
| Lipid lowering medication (ref: no) |  |  |  |  |
| Yes, lipid lowering medication | 1.54 (1.24–1.89) | 1.20 (0.71–2.32) | 1.59 (1.26–1.97) | 1.42 (1.07–1.90) |
| Predictive performance |  |  |  |  |
| AUC (95% CI) | 0.76 (0.74–0.78) | 0.89 (0.85–0.93) | 0.75 (0.73–0.77) | 0.82 (0.80–0.84) |
| TPR_FPR10%_ | 0.38 | 0.75 | 0.35 | 0.58 |

**Notes:** AOR: adjusted odds ratio. CrI: credible interval. CMD: cardiometabolic disease. BP: blood pressure. HDL-C: high-density lipoprotein cholesterol. Estimates are posterior median odds ratios with 95% credible intervals from regularised Bayesian logistic regression, adjusted for follow-up duration. Continuous predictors are scaled to clinically meaningful units: blood pressure per 10 mmHg, waist circumference per 5 cm, and HDL-C and non-HDL-C per 10 mg/dL. Analytical samples differed by outcome.

1 Any cardiometabolic disease: n=11,964 after excluding prevalent T2D, heart disease, or stroke and incomplete cases; n=722 incident cardiometabolic disease cases during follow-up (682 single, 40 multimorbidity).
2 Cardiometabolic multimorbidity: n=11,964 after excluding prevalent T2D, heart disease, or stroke and incomplete cases; n=40 incident multimorbidity cases during follow-up.
3 CVD (heart disease or stroke): n=12,456 after excluding prevalent heart disease or stroke and incomplete cases; n=538 incident CVD cases during follow-up.
4 T2D: n=12,680 after excluding prevalent T2D and incomplete cases; n=302 incident T2D cases during follow-up.

Table A10*.* Predictive performance of SCORE2 and the regularised Bayesian logistic regression model across cardiometabolic outcomes

| **Models** | **AUC (95% CI)** | **TPR_FPR 10%_** |
| --- | --- | --- |
| **CVD (heart disease or stroke)^1^** |  |  |
| Regularised Bayesian logistic regression | 0.75 (0.73–0.77) | 0.35 |
| SCORE2 | 0.72 (0.69–0.74) | 0.31 |
| **Any cardiometabolic disease^2^** |  |  |
| Regularised Bayesian logistic regression | 0.76 (0.74–0.78) | 0.38 |
| SCORE2 | 0.71 (0.69–0.73) | 0.31 |
| **Cardiometabolic multimorbidity^3^** |  |  |
| Regularised Bayesian logistic regression | 0.89 (0.86–0.93) | 0.75 |
| SCORE2 | 0.83 (0.78–0.88) | 0.48 |

**Notes**: AUC: area under the receiver operating characteristic curve. CI: confidence interval. TPR_FPR10%:_ sensitivity at a fixed false-positive rate of 10%. The regularised Bayesian logistic regression model refers to the primary prediction model developed in this study (main analysis). SCORE2 was calculated using the published algorithm with moderate-risk regional recalibration corresponding to Sweden’s risk region, based on age, sex, current smoking, systolic blood pressure, total cholesterol, and HDL-C. SCORE2 coefficients were applied directly without refitting. Comparisons for any cardiometabolic disease and cardiometabolic multimorbidity were exploratory because SCORE2 was developed to predict 10-year risk of fatal and non-fatal cardiovascular disease.

1 CVD (heart disease or stroke): n=12,456 after excluding prevalent heart disease or stroke and incomplete cases; n=538 incident CVD cases during follow-up.
2 Any cardiometabolic disease: n=11,964 after excluding prevalent T2D, heart disease, or stroke and incomplete cases; n=722 incident cardiometabolic disease cases during follow-up (682 single, 40 multimorbidity).
3 Cardiometabolic multimorbidity: n=11,964 after excluding prevalent T2D, heart disease, or stroke and incomplete cases; n=40 incident multimorbidity cases during follow-up.

Figure A1***.*** Sample flowchart

Notes:

*Total sample size for sensitivity analysis with multiple imputation.

**Total sample size for main analysis.

Figure A2. Calibration of cross-validated predictions for cardiometabolic disease and multimorbidity

**Notes**: Each panel shows the observed event rate against the predicted probability. Blue dots show the observed event rate within quantile bins of the predicted probability (10 bins for any cardiometabolic disease; 8 bins for cardiometabolic multimorbidity), with Wald 95% CI. The distribution of predicted probabilities is shown at the bottom of the graphs. The grey dashed line is perfect calibration (intercept = 0, slope = 1).

Panel metrics:

A) Any cardiometabolic disease (unregularised): intercept = -0.12 (95% CI -0.32–0.09), slope = 0.94 (95% CI 0.86–1.02);

B) Any cardiometabolic disease (regularised): intercept = -0.01 (95% CI -0.22–0.21), slope = 0.99 (95% CI 0.91–1.07);

C) Cardiometabolic multimorbidity (unregularised): intercept = -1.10 (95% CI -1.70–-0.50), slope = 0.70 (95% CI 0.53–0.86);

D) Cardiometabolic multimorbidity (regularised): intercept = 0.27 (95% CI -0.33–0.87), slope = 1.02 (95% CI 0.79–1.25).

Intercepts closer to 0 and slopes closer to 1 indicate better calibration.

Figure A3***.*** Calibration plot for regularised Bayesian with multiple imputation and Bayesian Additive Tree Regression (BART)

**Notes**:

Each panel shows the observed event rate against the predicted probability. Blue dots show the observed event rate within quantile bins of the predicted probability (10 bins for any cardiometabolic disease; 8 bins for cardiometabolic multimorbidity), with Wald 95% CI. The distribution of predicted probabilities is shown at the bottom of the graphs. The grey dashed line is perfect calibration (intercept = 0, slope = 1).

Panel metrics:

A) Any cardiometabolic disease (imputed) intercept = 0.01 (95% CI -0.06–0.08), slope = 0.99 (95% CI 0.92–1.08);

B) Any cardiometabolic disease (BART) intercept = -0.01 (95% CI -0.08–0.07), slope = 0.95 (95% CI 0.86–1.03);

C) Cardiometabolic multimorbidity (imputed) intercept = 0.15 (95% CI -0.13–0.43), slope = 1.02 (95% CI 0.82–1.23);

D) Cardiometabolic multimorbidity (BART) intercept = -0.04 (95% CI -0.31–0.24), slope = 0.81 (95% CI 0.61–1.01).

intercepts closer to 0 and slopes closer to 1 indicate better calibration.

Figure A4***.*** Estimated odds ratios (ORs) from regularised Bayesian logistic regression models for any cardiometabolic disease, cardiometabolic multimorbidity, heart disease or stroke, and diabetes

Notes: Points show posterior median odds ratios and horizontal lines show 95% credible intervals from regularised Bayesian logistic regression. Odds ratios are shown on a log scale. The vertical line indicates no association (OR = 1). Continuous predictors were scaled to clinically meaningful units, systolic blood pressure per 10 mmHg, waist circumference per 5 cm, and HDL-C and non-HDL-C per 10 mg/dL. Analytical samples differed by outcome as described in Figure A1.
